# The arousal effect of sugammadex reversal of neuromuscular blockade differs with anesthetic depth in propofol-remifentanil anesthesia: a randomized controlled trial

**DOI:** 10.1038/s41598-023-48031-6

**Published:** 2023-11-27

**Authors:** Jeayoun Kim, Jie Ae Kim, Jae Ni Jang, Mikyung Yang, Hyun Joo Ahn, Jiwon Choi, Sungwoo Jo

**Affiliations:** 1 Department of Anesthesiology and Pain Medicine, Samsung Medical Center, Sungkyunkwan University School of Medicine, 81 Irwon‑Ro, Gangnam‑Gu, Seoul, 06351 Korea; 2 Department of Anesthesiology and Pain Medicine, International St. Mary’s Hospital, Catholic Kwandong University School of Medicine, Incheon, Korea

**Keywords:** Outcomes research, Clinical trial design

## Abstract

Sugammadex reverses neuromuscular blockade by encapsulating steroidal neuromuscular blockers; therefore, it does not pharmacologically affect sedation levels. However, some clinicians avoid using it because of sudden unwanted acting out or patient arousal. Previous studies suggested sugammadex-induced awakening, but frontal muscle contraction after sugammadex administration compromised reliability of results obtained from EEG-based anesthesia depth monitoring tools like bispectral index (BIS). We hypothesized that sugammadex would affect patients’ arousal depending on their baseline levels of sedation. We evaluated arousal signs after sugammadex administration with BIS between 25 − 35 and 45 − 55 under steady-state propofol-remifentanil anesthesia at the end of a surgery (n = 33 in each group). After sugammadex administration, twelve patients with a BIS of 45 − 55 showed clinical signs of awakening but none with a BIS of 25 − 35 (36.4% vs. 0%, *P* = 0.001). The distribution of the modified observer’s assessment of alertness/sedation scale scores was also significantly different between the two groups (*P* < 0.001). Changes in the BIS were significantly greater in the BIS 45 − 55 than in the 25 − 35 group (median difference, 7; 95% CI 2 − 19, *P* = 0.002). Arousal after sugammadex was affected by patient sedation levels, and clinical signs of awakening appeared only in those with BIS 45 − 55. Unwanted arousal of the patient should be considered when using sugammadex under shallow anesthesia.

**Clinical trial registry number:** Clinical Trial Registry of Korea (https://cris.nih.go.kr; Principal investigator: Jieae Kim; Registration number: KCT0006248; Date of first registration: 11/06/2021).

## Introduction

Sugammadex is a modified γ-cyclodextrin, which promptly reverses the steroidal nondepolarizing neuromuscular blocking agents. Although previous studies have reported a sudden increase in bispectral index (BIS) values after sugammadex reversal of neuromuscular blockade, many studies have supported that BIS increases are likely to be influenced by increased electromyography (EMG) activity following sugammadex administration, rather than true arousal^1–4^. However, unexpected and sudden arousal after sugammadex use baffles clinicians, and further investigation of the effect of sugammadex on arousal is required. Recent studies have reported that sugammadex can encapsulate other drugs, such as propofol and remifentanil, in addition to non-steroidal neuromuscular blockers^5^. Another study reported that low dose sugammadex administration (2 mg kg^−1^) provoked a lighter anesthesia depth pattern characterized by an increase in beta activity and a decrease in delta activity, without clinical signs of awakening when administering sugammadex in deep sevoflurane anesthesia^6^. Some studies have suggested that the weak central effect of afferentation induced by sugammadex makes it difficult to quantify arousal at deeper levels of hypnosis^4, 7^.

This study hypothesized that the arousal effect of sugammadex is affected by the underlying degree of sedation. In contrast to prior research that relied on the BIS to assess the arousal effect of sugammadex, which might be disputed because of possible EMG-driven BIS elevation, we focused on the clinical signs of awakening as our primary outcome. We compared the clinical signs of arousal after sugammadex administration between BIS 25 − 35 and 45 − 55 at a steady state using a continuous infusion of propofol and remifentanil.

## Methods

### Study design

This was an investigator-initiated, single-center, randomized, patient-blinded trial with a two-arm, parallel design. The Institutional Review Board of Samsung Medical Center approved this study (no. SMC 2021-04-124, approval date: 21/05/2021). This study was registered with the Clinical Trial Registry of Korea prior to the recruitment of the first participant (https://cris.nih.go.kr; principal investigator: Jie Ae Kim; Registration number: KCT0006248; Date of first registration: 11/06/2021). Written informed consent was obtained from all participants before enrolment. All the experiments were performed in accordance with the Declaration of Helsinki.

### Study population

Between November 2021 and July 2022, all consecutive patients scheduled for major abdominal surgery taking 1 h or more under propofol-remifentanil intravenous anesthesia, were screened, and patients who met the inclusion criteria were contracted by primary investigators a day before the surgery to obtain written informed consent.

The inclusion criteria were age between 19 and 75 years and American Society of Anesthesiologists physical status I or II. The exclusion criteria were as follows: emergency surgery; body mass index < 18.5 kg m^–2^ or > 35 kg m^–2^; presence of severe kidney, liver, neuromuscular, central nervous system, psychiatric, or metabolic disease; drug or alcohol abuse; pregnant or nursing women; and allergies or a history of hypersensitivity to the study drug. Patients who withdrew consent or had BIS values outside the target range at the time of sugammadex administration were excluded after allocation.

### Randomization and blinding methods

Computer-generated randomization was performed by principal investigator at a 1:1 ratio with permuted block design with a block size of four through an online application accessed from the website, “Sealedenvelope.com”. Eligible patients were randomly assigned to either the deep or shallow anesthesia group according to a randomization list. The attending anesthesiologists were informed of the group allocation immediately before anesthesia induction using the sealed opaque envelope technique. The patients and investigators involved in the postoperative follow-up and data analysis were blinded to the group allocation.

### Intervention

Patients were randomly allocated to two groups: deep and shallow anesthesia, at the completion of the operation. The effect-site concentration (Ce) of propofol were adjusted to maintain BIS values between 25 − 35 in the deep and 45 − 55 in the shallow anesthesia group for 10 min of the “stabilization period.” The Ce of remifentanil was fixed at 2 ng mL^–1^ in both groups. Once a steady state of propofol-remifentanil anesthesia was achieved and deep NMB (a train of four [TOF] count 0 and post-tetanic count [PTC] ≥ 1) was confirmed, 4 mg kg^–1^ of sugammadex was injected (baseline, T_0_)^8^. Before sugammadex injection, the Analgesia-Nociception Index (ANI) values at T_0_ were recorded. The Ce of propofol and remifentanil at steady state was kept constant during the 5 min study period (T_5_). The outcome variables were recorded every minute.

### Outcomes

The primary outcome was the incidence of clinical signs of awakening (eye opening, spontaneous movement, cough, response to simple orders, or complete recovery) during the 5 min study period after the administration of sugammadex in steady-state propofol-remifentanil anesthesia^4^. Secondary outcomes were the Modified Observers Assessment of Alertness and Sedation Scale (MOAA/S scale)^9^, BIS scores, and EMG values from the BIS monitor. The EMG values of the BIS monitor represented the average total power within the 70 − 100 Hz frequency range over the previous 10 s, and were presented on a logarithmic scale.

### Anesthesia and patient monitoring

Standard monitoring, including pulse oximetry, three-lead electrocardiography, and noninvasive blood pressure measurements, was performed. A BIS sensor (BIS Quatro™; Medtronic, Dublin, Ireland) was attached to the forehead and connected to BIS monitor (BIS complete monitoring system; Medtronic, Dublin, Ireland) according to the manufacturer’s instructions as we described elsewhere^10^. After we confirmed the signal quality index was above 95%, BIS values were recorded from the time of anesthesia induction until the end of the study. We applied a TOF-Watch SX (Organon Ltd., Dublin, Ireland) to the right or left adductor pollicis to monitor NMB. The TOF-Watch SX was calibrated using the automated the CAL2 mode. Anesthesia was induced and maintained using target-controlled infusion (TCI) of propofol and remifentanil with a continuous infusion device (Orchestra®; Fresenius Vial, Brezins, France). In the TCI system, propofol and remifentanil were administered using the Schnider`s and Minto model, respectively. The patient’s trachea was intubated following administration of 0.7 mg kg^–1^ of rocuronium and a TOF count of 0. The depth of anesthesia was adjusted to maintain a BIS between 40 and 60. We maintained a TOF count of zero throughout the surgery. Mechanical ventilation was adjusted to maintain normocarbia (35–40 mmHg end-tidal carbon dioxide) using a mixture of oxygen and medical air (fraction of inspired oxygen: 0.5). The ulnar nerve was stimulated supramaximally through surface electrodes in the TOF mode every 15 s. The ANI (MetroDoloris Medical Systems, Lille, France) was monitored using the proprietary module available for the Root® monitoring platform (Masimo, Irvine, CA, USA), which continuously displays an average measurement of ANI made over the previous 64 s (instantaneous ANI, ANIi) and 240 s (mean ANI, ANIm). After we confirmed the reversal of NMB after the completion of the study (TOF ratio ≥ 90%), propofol and remifentanil were discontinued, and 0.01 mg kg^–1^ hydromorphone was administrated for pain control. Incomplete reversal of NMB at T_5_ (TOF ratio < 90%) was treated with an additional 2 mg kg^–1^ sugammadex. In all but three patients (three and none in the deep and shallow anesthesia groups, respectively), NMB was completely reversed after the administration of 4 mg kg^–1^ sugammadex; therefore, an additional dose of 2 mg kg^–1^ sugammadex was not required.

After the trachea was extubated, the patients were transferred to the post anesthesia care unit. The patients were interviewed using a modified version of the Brice questionnaire for intraoperative recall after returning to a fully alert status^11, 12^. Patients were followed up for 24 h after surgery for any adverse events in relation to this study.

### Statistical analysis

Power calculations were performed based on the primary endpoints. According to our preliminary study that included 24 patients, the incidence of clinical signs of awakening was 25% in the shallow anesthesia group (BIS 45 − 55) and 0% in the deep anesthesia group (BIS 25 − 35). With a two-tailed significance level of 0.05 and a power of 80%, 62 patients were required. Considering a dropout rate of 5%, 66 patients were in the study.

Categorical variables were presented as numbers and percentages (%). Continuous variables were presented as mean ± standard deviation (SD), when the data were normally distributed, or as median (interquartile range, IQR) when the distribution was skewed. Normality was tested using the Shapiro–Wilk test. Categorical variables (clinical signs of awakening and MOAA/S scale score) were analyzed using the chi-square or Fisher’s exact test. We compared the change in BIS values from T_0_ to BIS_max_ between the two groups after sugammadex injection using the *t* test or Mann–Whitney *U* test. In addition, we compared the baseline and maximum values of BIS and EMG after sugammadex injection using a paired *t* test or Wilcoxon signed-rank test. The BIS values were recorded every 1 min for 5 min after sugammadex administration, and the patterns of change were compared between the deep and shallow anesthesia groups using a generalized estimating equation (GEE). Statistical analyses were performed using SPSS (version 27.0; IBM Corp., Armonk, NY, USA). We considered *P* values less than 0.05 statistically significant.

## Results

**Figure 1:**
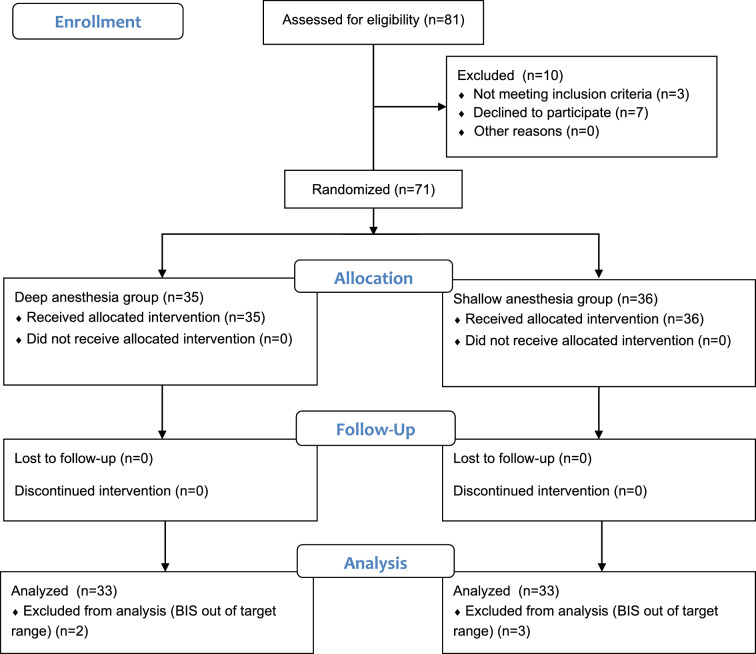
CONSORT diagram.

Between November 8, 2021, and July 28, 2022, 81 patients were assessed for eligibility, and 66 patients were enrolled for this study. Data from the final total of 66 patients were analyzed (Fig. 1).

Table 1 presents the baseline characteristics and intraoperative data. At the time of sugammadex administration (T_0_), the Ce of propofol was significantly higher in the deep anesthesia group than in the shallow anesthesia group. Instantaneous and mean ANI were not significantly different between the two groups at T_0_. The median (IQR) time from T_0_ to the time of TOF ratio ≥ 90% was not significantly different between the two groups.

**Table 1:** Baseline characteristics and intraoperative data.

Variable	Deep anesthesia group	Shallow anesthesia group	*P* value
	(N = 33)	(N = 33)	
Male	18	18	> 0.99
Age (yr)	59.4 (10.1)	56.4 (10.9)	0.255
Body mass index (kg/m^2^)	23.8 (2.6)	23.7 (2.7)	0.89
ASA I/II	11/22	11/22	> 0.99
Duration of surgery (min)	142 [116, 178]	136 [114, 182]	0.798
Duration of anesthesia (min)	185 [160, 236]	187 [158, 228.5]	0.99
Effect site concentration of propofol at T_0_ (µg/mL)	4.6 (1.1)	2.7 (0.7)	< 0.001
Analgesia-nociception index at T_0_			
Instantaneous ANI	60.4 (14.9)	62.5 (13.0)	0.517
Mean ANI	62.2 (15.3)	64.2 (12.9)	0.547
Post tetanic count at T_0_	6 [1.5, 9]	6 [3, 8.5]	0.771
Total amount of rocuronium	110 [80, 130]	82.5 [75, 100]	0.835
Total amount of sugammadex	260 [220, 300]	240 [220, 275]	0.382
Time to TOF ratio 0.9 (min)	4 [3.5, 5]	5 [4, 5]	0.137
Time to maximum BIS (min)	3 [1, 5]	4 [3, 5]	0.146

Data expressed as mean (SD), median [IQR], or n (%).

*ASA* American Society of Anesthesiologists’ physical status, *T0* at the time of sugammadex administration, *ANI* analgesia-nociception index, *TOF* train of four, *BIS* bispectral index.

**Figure 2:**
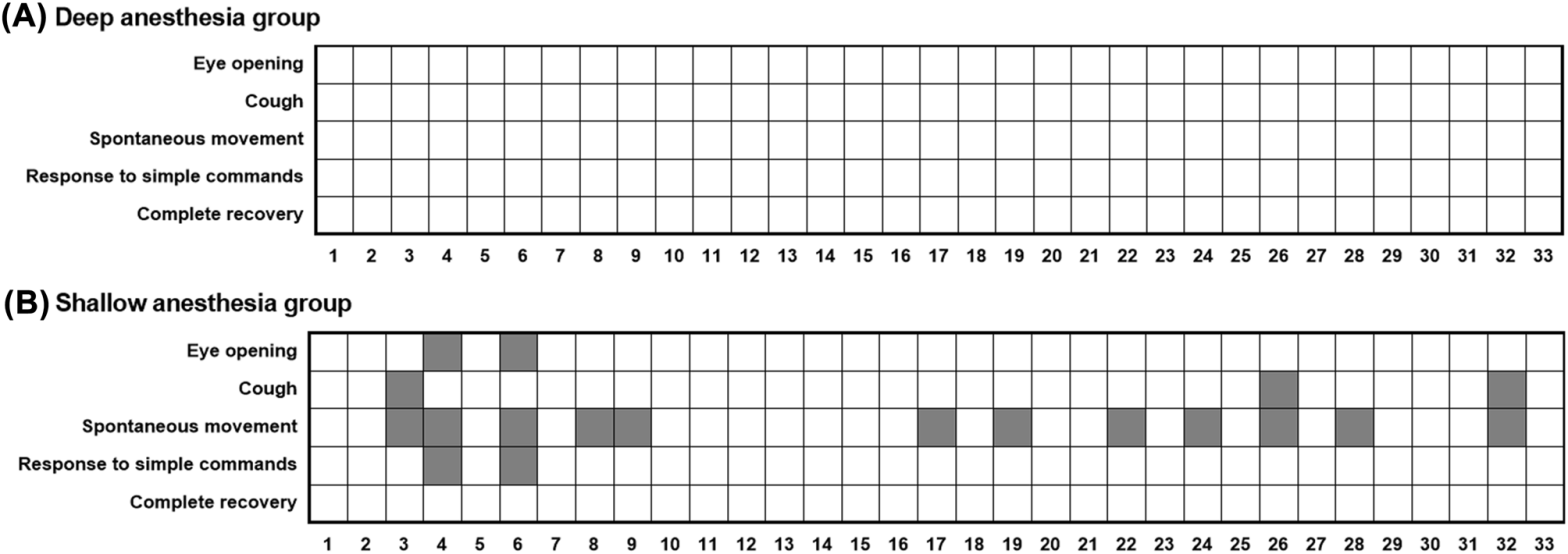
Clinical signs of awakening which occurred in the shallow and deep anesthesia groups.

**Figure 3:**
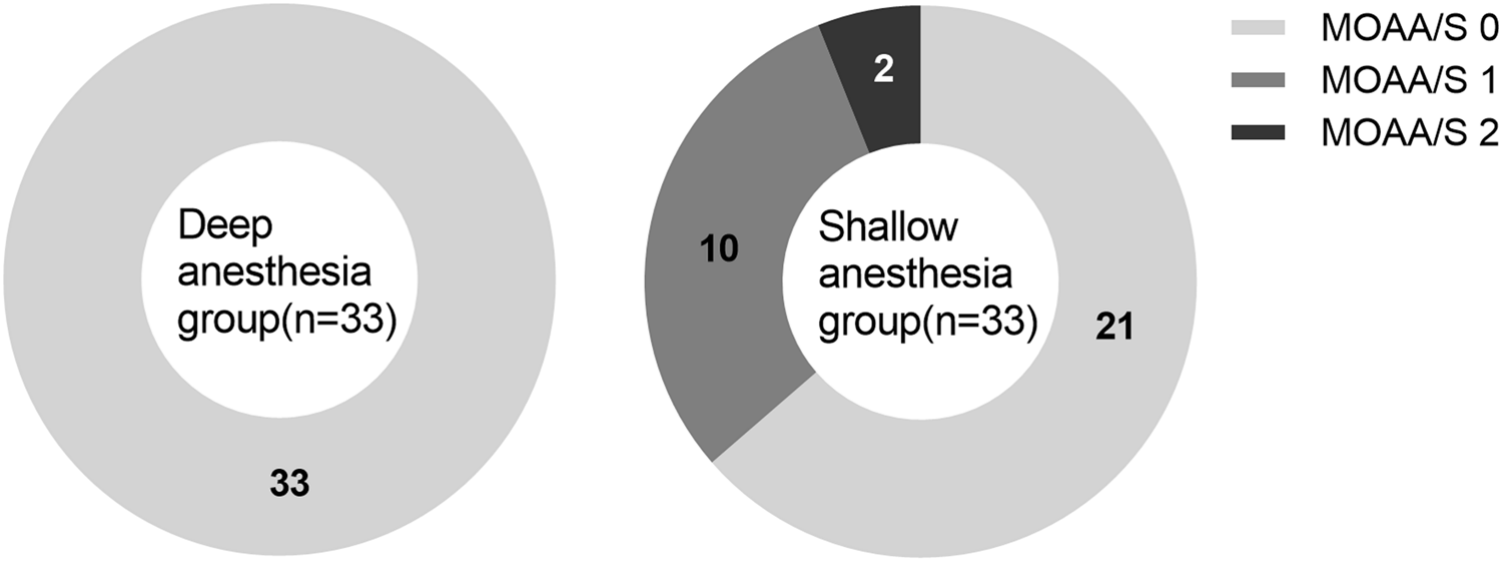
Modified observer’s assessment of alertness and sedation (MOAA/S) score in the shallow and deep anesthesia group. The distributions of the MOAA/S score during the study period were significantly different between the two groups (*P* < 0.001).

Clinical signs of awakening occurred only in the shallow anesthesia group (12/33, 36.4% vs. 0/33, 0%; *P* = 0.001). Twelve patients showed spontaneous movements including grimaces and limb, head, neck, and shoulder movements. Two patients demonstrated eye opening, followed simple orders, and responded with mild prodding or shaking (Fig. 2). The distribution of MOAA/S scores during the study period differed between the two groups (*P* < 0.001) (Fig. 3).

**Figure 4:**
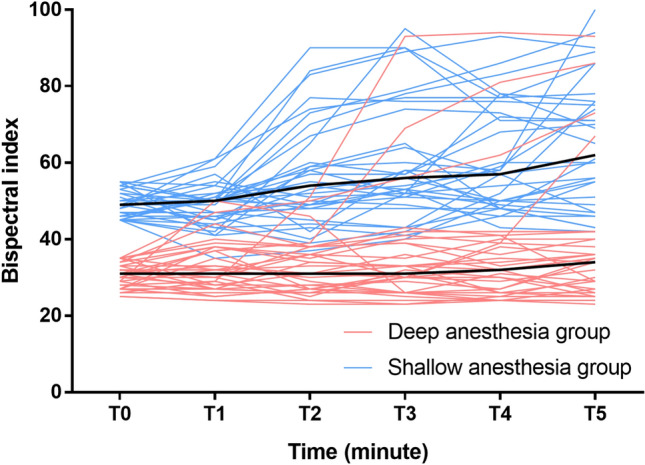
Bispectral index (BIS) values of the individual patients (thin lines) and median BIS values of each group (thick lines) during the 5 min study period. Sugammadex was given at time point T_0_. The pattern of changes in BIS values over time compared by the generalized estimating equation was significantly different between the two groups (*P* = 0.024).

The BIS and EMG values obtained from the BIS monitor at T_0_ and their maximum values during the study period are presented in Table 2. Despite maintaining the steady state of propofol-remifentanil, BIS levels increased significantly with sugammadex injection in both groups (median increase, 18; 95% confidence interval CI 11 − 24; *P* < 0.001 and 15; 95% CI 10.5 − 20; *P* < 0.001 in the shallow and deep anesthesia groups, respectively). The change in BIS values from T_0_ to BIS_max_ was significantly greater in the shallow anesthesia group than in the deep anesthesia group (median difference, 7; 95% CI 2 − 19; *P* = 0.002). Change in the EMG values from T_0_ to their maximum values was similar (median difference, 7; 95% CI 1 − 14; *P* = 0.002) (Table 2). The changes in BIS values following the administration of sugammadex are depicted in Fig. 4. According to the GEE model, at equivalent time points, patients in the deep anesthesia group exhibited significantly lower BIS values compared with those in the shallow anesthesia group, with an estimated difference of 16.7 (95% CI 14.2–19.2, *P* < 0.001). Following the administration of sugammadex, on average, the BIS value increased by 1.3 (0.2–2.5) units for every min that elapsed in the deep anesthesia group and 3.2 (2.1–4.2) units in the shallow anesthesia group. The rate of increase is significantly higher in shallow anesthesia group (*P* = 0.024).

**Table 2:** Comparison of BIS and EMG values before and after the administration of sugammadex.

	Deep anesthesia group		Shallow anesthesia group		*P* value
	Baseline	Max after sugammadex	Baseline	Max after sugammadex	
BIS	31 [27.5, 33]	35 [31, 42]*	49 [46, 53]	64 [56, 82]*	0.002
EMG	26 [25, 27]	29 [26, 36.5]*	26 [25.5, 27]	39 [29, 53.5]*	0.002

Data expressed as median [IQR].

*P*-value for the maximum change in BIS or EMG values after sugammadex administration between the deep and shallow anesthesia groups.

*BIS* bispectral index, *EMG* electromyography from the BIS monitor, *Max* maximum of value of each parameter after sugammadex administration.

*P < 0.001 compared with the corresponding baseline value.

No patient had postoperative residual NMB and none experienced an explicit recall or adverse effects in relation with this study.

## Discussion

In this randomized controlled trial, sugammadex reversal of NMB increased BIS in both shallow and deep anesthesia groups despite maintaining a steady state of propofol-remifentanil infusion and had a greater influence on the arousal in shallow than in deep anesthesia group. Previous studies only used the BIS as a measure of awakening after sugammadex administration, which might be disputed because BIS values are falsely increased by an activated EMG signal^1–4^. Instead, we used clinical signs of awakening as the primary outcome and those that occurred only in the shallow anesthesia group in the current study. Some patients responded to mild prodding and shaking (MOAA/S score of 2) and demonstrated apparent awakening (eye opening and response to simple orders). In addition, the magnitudes of increase in the BIS and EMG values were significantly greater in the shallow anesthesia group than in the deep anesthesia group.

The differential influence of sugammadex reversal of NMB with the anesthetic depth could be explained by “afferentation.” Unexpected movements and increased BIS could be considered an evidence of awakening induced by an increased afferent signal, as in previous studies^4, 13–15^. Muscle stimulation caused by certain agents or maneuvers activates stretch-responsive sensory receptors (muscle spindles) and generates action potentials. These action potentials result in afferent inputs to the brain-awakening centers, ultimately leading to cortical arousal^16, 17^. When the patients are paralyzed with neuromuscular blocking agents, the muscle response to external stimuli is eliminated and the afferent signal generated in muscle stretch receptors decreases, resulting in reduced EEG responses. However, once these patients are reversed by sugammadex, a sudden increase in afferent signals is generated in muscle stretch receptors and transmitted to the arousal center of the brain via afferent nerve pathways, which can provoke arousal^16–18^. In this regard, the intensity of afferent signals activated by sugammadex-mediated reversal of NMB and the depth of hypnosis at the time of sugammadex administration may be important factors for arousal in the central nervous system. If the activated muscle mass was not large enough to induce arousal, sugammadex administration would not produce arousal. In contrast, if the NMB was deeper, the probability of awakening was higher^4, 7^. If the anesthetic depth at the time of sugammadex administration was too deep to be affected by afferent signals, sugammadex would not induce arousal that could be measured in the BIS or clinical signs of awakening^4, 7^. Since we balanced the depth of NMB in both groups, aiming for a TOF of 0 and a PTC of 1 or more, the cause of the differential effect in the current study could be explained by the latter.

Binding between sugammadex and propofol may serve as an alternative pharmacokinetic mechanism that contributes to the differential effects of sugammadex based on the level of sedation. This possibility was previously dismissed because of the low affinity between the propofol and sugammadex molecules; nevertheless, it has recently regained attention. Computer modeling and ex vivo brain-slice data indicated the possibility of a decrease in free propofol concentration within the central compartment due to the binding between propofol and sugammadex in the presence of excess free sugammadex. This suggests that a potential modest reduction in the effects of propofol could not be completely ruled out^5^. However, these data were based on computer modeling and brain slice experiments, and we could not assert a clinical impact.

The clinical features that were considered signs of awakening in the current study are disputable in that the movements could be unmasked spinal withdrawal reflexes to noxious stimuli. A relatively shallow level of hypnosis could not suppress the reflex response to noxious stimuli caused by the endotracheal tube or surgical site pain^19^. To minimize nociceptive reflexes, we attempted to keep the patients painless. External stimulation was not permitted and remifentanil was maintained at a Ce of 2 ng mL^–1^ during the study period, which was sufficient to prevent laryngeal irritation^20, 21^. We also used ANIs as surrogates for pain and maintained ANIs above 50 in both groups at a steady state, which showed a high negative predictive value for postoperative pain^22–24^. Under these conditions, we observed signs of a return of consciousness (eye opening and response to simple orders). In addition, spinal cord reflexes would appear and disappear rapidly, within 0.5 s, to noxious stimuli. These signs cannot be explained solely by nociceptive reflexes.

We demonstrated that if the depth of hypnosis was relatively shallow, even within the range of general anesthesia, sugammadex reversal of NMB could trigger clinical signs of awakening. Under normal circumstances, in which sugammadex is administered for tracheal extubation at the end of surgery, this reversal is not problematic. However, if a clinical need for sudden reversal of NMB arises during surgery, these signs may have clinical implications. For example, intraoperative neuromonitoring may be required in thyroid, neuro, ear, or thoracoabdominal aortic aneurysm surgery, in which clinicians may use sugammadex to resume normal muscle twitch activity^25, 26^. If the depth of hypnosis is relatively shallow, unexpected movement can occur after sugammadex administration, which may cause a return of consciousness and unwanted harm to the patient.

Our study had several limitations. First, this study was conducted at a single center; therefore, the generalizability of our findings is limited, and external validation is required. Furthermore, our explanations of the results of this study are based on the assumption that awakening due to increased afferent signals is the main cause of unexpected movements after sugammadex administration. However, based on our study design, we could not clarify the cause of the clinical signs of awakening after sugammadex administration. In addition, BIS values between 45 and 55 do not necessarily indicate an adequate depth of anesthesia^27^; patients may have been awake at the time of sugammadex administration. These patients showed signs of awakening only after recovery from NMB. Further research is warranted to evaluate raw EEG patterns before and after sugammadex administration. Finally, BIS values between 25 and 35 may be ethically questionable due to concerns regarding postoperative delirium. However, this range was temporarily maintained at the end of the surgery. Furthermore, a recent large randomized controlled trial demonstrated that reducing the median cumulative time with EEG suppression and BIS < 40, as achieved by EEG guided anesthesia, did not result in a decrease in delirium incidence among elderly patients^28^. In this regard, we assumed that the brief period of deep anesthesia in our study would not pose significant harm to the study participants.

## Conclusion

Sugammadex reversal of NMB resulted in a higher incidence of clinical signs of awakening and a greater increase in BIS during shallow than during deep anesthesia.

## Data Availability

The dataset generated during the current study is available from the corresponding author upon request.
